# Innovative health financing for refugees

**DOI:** 10.1186/s12916-018-1068-9

**Published:** 2018-06-15

**Authors:** Paul Spiegel, Rebecca Chanis, Antonio Trujillo

**Affiliations:** Johns Hopkins Bloomberg School of Public Health and Center for Humanitarian Health, 615 N. Wolfe St, Baltimore, MD 21205 USA

**Keywords:** Refugee, Financing, Humanitarian, Health systems

## Abstract

**Background:**

More than 65 million persons are currently forcibly displaced, of whom more than 22 million are refugees. Conflicts are increasing, and existing ones are becoming more protracted; a refugee remains a refugee for more than 10 years. Funding for refugee assistance comes primarily from high-income countries after an emergency has occurred. The United Nations High Commissioner for Refugees spent approximately 12% of its budget on health, nutrition, food security, water, and sanitation in 2016. The current modalities used to fund refugee emergencies are not sustainable and will worsen as health needs increase and health services become more expensive, particularly in middle-income countries.

**Main idea:**

Given the current number of complex conflicts and the magnitude of displacement, new sources of funding and innovative financing instruments are needed. This article explores diverse sources of innovative humanitarian health financing for refugees. Ultimately, the goal is to integrate refugees into a host country’s functioning national health system, which, if done thoughtfully, should improve health services and outcomes for both nationals and refugees. Addressing the increasing level of humanitarian needs for refugees requires a wide range of resources and a sophisticated financing toolkit that can be adapted to different refugee contexts. Improving health financing for refugees requires a paradigm shift towards pre-emergency and multi-year planning using risk-transfer instruments. It necessitates a wide range of public and private partners and varied resources that range from health insurance, bonds, and concessional loans to host countries with innovative methods for purchasing projects and services such as pay for performance. These modalities need to be employed according to specific refugee contexts, and the potential risks must be considered carefully.

**Conclusion:**

We propose the exploration of a Refugee Health Financing Model, or FinRef, for the acute phase of an emergency, and different forms of health insurance as well as pay-for-performance modalities in protracted settings. Such innovations will require traditional and non-traditional partners to work together to trial different financial schemes. Donors and investors need to be prepared to experiment and accept failure of some models in certain contexts. Ultimately, different innovative financing models will be able to provide more sustainable and effective health services to refugees and their host populations in the near future.

## Background

By the end of 2016, there were 65.6 million forcibly displaced persons globally, of whom 22.5 million were refugees [1]. Whether refugees live in camps or are integrated into host populations, and whether they are settled in low-income countries (LICs) or middle-income countries (MICs), governments often struggle to meet the health needs of these populations. Host countries’ existing health systems are often weak, and the added burden of providing for refugees can make them even more fragile.

The ultimate goal is to have a health care system for refugees that is integrated into a functioning national system [2]; if implemented thoughtfully, this integration should benefit the refugees and the host populations. However, if national health systems are not functioning or those systems are overwhelmed, particularly at the beginning of an acute emergency, then parallel systems may need to be established.

The paper is premised on five declarations: (1) Refugees, like all other persons in the world, have a right to universal health care coverage; (2) The humanitarian system is currently overstretched and underfunded, and it cannot meet the current demands of multiple and increasingly protracted humanitarian emergencies [3]; (3) Traditional funding for humanitarian emergencies is insufficient, unsustainable, and predominantly provided by high-income countries (HICs); (4) Current funding instruments overwhelmingly consist of post-emergency external assistance provided to United Nations (UN) and international non-governmental organizations (NGOs); and (5) Refugee crises are generally protracted, rather than short term; the average refugee remains a refugee for more than 10 years [4].

Refugee contexts and their various attributes can be categorized in numerous ways. For this paper, we use the following framework (Table 1). How and what type of refugee health care is established depends upon the contexts listed in Table 1. For example, types of services and their quality may differ between the acute emergency phase, where there is often limited capacity and security, compared with the protracted phase, where there is more stability. Parallel health systems are often established in camp settings compared with out-of-camp settings, where they are often integrated within existing national systems. Types of services and ability to refer may differ between urban/peri-urban and rural settings as well as between LICs and MICs. Although it is difficult to clearly define functioning and non-functioning district health systems, the essential issue relates to the ability of the district health services to integrate refugees into an existing system that will provide sufficient access and quality of services. If such a system cannot do this, even with support from international organizations, then alternatives need to occur, such as providing parallel services by NGOs or the private sector. However, such parallel services should be avoided if possible.

**Table 1:** Refugee contexts framework

Phase	Location	Host income level	District health system
● Preparedness (pre-emergency) ● Acute emergency ● Protracted (> 5 years) ● Durable solutions o Voluntary repatriation o Local integration o Resettlement	● Camp, out of camp ● Urban, rural	● Low-income country (LIC) ● Middle-income country (MIC)	● Functioning ● Semi-functioning ● Non-functioning

## Instruments for financing humanitarian emergency risk

Available financing instruments have two fundamental components: risk and timing [5]. Risk is defined as the potential for or probability of a loss and can be related to individuals or events. Risk-retention tools hold refugee host countries responsible for risk. They provide for more flexible payments, as they can be spent at their discretion. These tools include contingency funds, budget allocations, contingent credit, budget reallocations, tax increases, and post-emergency credit. Risk-transfer tools allow host countries to transfer risk to another party. This provides more security by having another entity be responsible for the risk. These tools include insurance, reinsurance, bonds, swaps, and donations.

Timing, the other essential component, relates to when the risky outcome occurs. Pre-emergency (ex ante) instruments depend upon planning for emergencies and include reserves, contingency funds, budget contingencies, contingent debt facilities, and risk-transfer products. Post-emergency (ex post) instruments do not depend upon planning for emergencies and include donations, budget reallocation, loans, and tax increases.

There are a variety of financing instruments available for preparing and responding to humanitarian emergencies that combine different features of timing and risk. Table 2 shows refugee humanitarian financing instruments categorized according to risk and time.

**Table 2:** Refugee humanitarian financing instruments listed according to risk and time

	Dependent upon planning	Not dependent upon planning
*Risk retention* (refugee host countries are responsible for risk)	● *Domestic contingency funds or budget allocations*: money for emergency relief set aside prior to event ● *Taxes and subsidies* to alter incentives for providing funding ● *Line of contingent credit*: a loan disbursed under certain circumstances	● *Budget reallocation* ● *Tax increases* ● *Post-emergency credit* ● *User fees* ● *Taxes and subsidies* to alter incentives for providing funding ● *Tariffs or subsidies* to alter prices of goods during emergencies
*Risk transfer* (refugee host countries transfer risk to another entity)	● *Traditional insurance or reinsurance*: contract where insured pays insurer a premium, and insurer agrees to pay for pre-specified and post-verified losses ● *Indexed insurance*: insurance contract where insurer makes payments based on certain external, measurable parameters or index ● *Capital market instruments*: financial instruments that can be bought or sold on capital markets, and investors shoulder risk (e.g., catastrophe bonds and swaps, Pandemic Emergency Financing Facility) ● *Contingency pooled UN funds* (e.g., Central Emergency Relief Fund and Country-Based Pooled Funds)	● *Discretionary post-emergency aid*: includes in-kind and cash transfers
		*Discretionary post-emergency aid is the most common instrument for aid delivery in humanitarian emergencies and is provided primarily by HICs*

## Traditional humanitarian and refugee financing

International humanitarian assistance reached a record high of US$27.3 billion by the end of 2016 (this included assistance related to conflict and natural disasters, refugees, internally displaced persons, and non-displaced persons). Humanitarian funding is mostly provided from a donor to an organization for either direct implementation or to be passed on to implementing partners. In 2016, nearly half of all international humanitarian assistance, primarily from government donors from the Organisation for Economic Co-operation and Development, was provided in the first instance to multi-lateral organizations, primarily to UN organizations. Five government donors (all HICs except for Turkey) contributed 65% of the total humanitarian funds in 2016, with the USA providing 31% and European countries combined providing 53%. As funding from some government donors slowed, the potential of funding from private sources (i.e., individuals, trusts and foundations, and companies) continued to increase to $6.9 billion (25%) [6].

Five crises in 2016 (in Syria, Yemen, South Sudan, Iraq, and Ethiopia) accounted for more than half (53%) of all funding allocated to specific emergencies. Protracted crises continue to absorb the largest volumes of international humanitarian assistance, yet much of the funding provided to these countries in protracted crises still arrives annually rather than in multi-year grants [6].

Protecting and assisting refugees is primarily the responsibility of the host state. Domestic governments often provide significant funding to the hosting of refugees. However, there are no standardized reporting systems, and thus financial contributions are difficult to estimate. The majority of refugees are hosted in countries with limited domestic capacity to support them. Thus, traditional refugee funding comes from risk-transfer instruments primarily by donations from HICs. The United Nations High Commissioner for Refugees (UNHCR) expenditures increased from $1.9 billion in 2012 to $3.2 billion in 2016 with Africa receiving $1.0 billion (31%) followed by the Middle East and North Africa with $0.9 billion (27%). Health, nutrition, food security, water, and sanitation were approximately 12% of the overall UNHCR’s 2016 expenditures. UNHCR receives the large majority of its funding from HICs [7].

## Innovative refugee health financing mechanisms

Innovative financing mechanisms are defined as non-traditional applications of overseas development assistance, joint public-private mechanisms, and flows that fundraise by tapping new and varied resources that deliver new financial solutions to humanitarian and/or development contexts [8]. Addressing the increasing level of humanitarian needs for refugees requires a wide range of resources and a sophisticated financing toolkit that can be adapted to different refugee contexts (Table 1). Improving health financing for refugees requires a paradigm shift towards pre-emergency and multi-year planning using risk-transfer instruments (Table 2). The following sections examine current and evolving innovative health financing mechanisms from various settings and then makes recommendations for refugee contexts.

### Insurance and bonds

#### Traditional health insurance

Insurance companies pool risk by having the insured pay premiums to the insurer. Should any insured entity suffer a loss, the insurance company will cover them. Insurers often buy reinsurance from a third party. Reinsurance shares risks and gains and reduces loss in the case of an extreme event for which an insurer cannot pay. A government or organization that insures humanitarian emergencies needs to determine how much risk it retains and how much it transfers, or whether it would just buy an insurance policy from a private company. There are various types of insurance schemes, from those that are publicly funded through some form of taxation (public insurance) to privately funded types (private insurance). Types of enrollment (mandatory, voluntary), contributions (income-based, community-based, risk-based), and management (public, non-profit, for-profit commercial, non-profit community) vary accordingly.

The main objective of refugee health insurance should be to integrate refugees into existing national systems, if they exist and are functioning. When such systems are “semi-functional,” external financial assistance and expertise may help some national systems improve sufficiently to provide health services for their own citizens and refugees. Numerous countries in Africa have integrated universal health coverage into their national frameworks, but progress towards implementation has been uneven [9]. In the future, as more countries in Africa provide universal health coverage, the more feasible it will be for refugees to integrate into such systems. In many areas where refugees are residing, national social welfare systems, including health insurance, are not available, and thus this would not be an option for refugees.

In protracted settings, when the health situation is relatively stable, traditional health insurance for refugees should be considered [10]. The majority of refugees currently live in protracted settings. However, relatively few refugees currently have access to public health insurance schemes. For health insurance for refugees to be feasible and sustainable, however, refugees must have access to livelihoods to pay for their premiums and co-share costs. The issue of livelihoods is complex and will not be discussed in detail here. However, the right to work for refugees is essential to reduce refugee dependency as well as the amount of donor assistance. The World Bank’s 2016 report entitled “Forcibly displaced: towards a development approach supporting refugees, the internally displaced, and their hosts” shows that refugee influxes often benefit the local economy, although who benefits within that community is more nuanced [4].

There are numerous direct and indirect benefits in allowing refugees to access national health insurance schemes. Improved access to health services and financial protection are clearly the two largest benefits. Indirect benefits include the provision of an official piece of documentation (a health insurance card) that may protect refugees from harassment by authorities and provide refugees with a sense of belonging and security, or allow them to send and receive remittances (Box 1). More data about refugees may be provided to UNHCR and its partners to more objectively decide who is most vulnerable. Other data can be collected from health insurance companies about who uses which services, where, and for what reason. The protection benefits and data may also allow for improvement in other sectors and programs. Although equity is an important component in health care, it must be one of many essential factors to be considered in providing health insurance. While a scheme may exclude a group of especially vulnerable refugees or those with specific illnesses, it may still be cost effective for some or the majority of refugees who have the possibility of paying health insurance premiums [10].

There will always be vulnerable populations in all societies who cannot afford to pay for health insurance. Decisions as to who is vulnerable and who will help to pay (fully or partially) for these vulnerable persons will need to be made. Depending upon the number of refugees contributing to the national system, the risk pool may have sufficiently grown to allow for subsidizing the insurance premiums and co-payments for these refugees as occurs with nationals. Other sources of revenue could come from UNHCR, which is currently funding millions of dollars in health care services via governments, NGOs, and faith-based organizations, often providing parallel services.

Finally, the provision of private health insurance is also a possibility, but it is nearly always significantly more expensive than national health insurance and should be avoided except in atypical circumstances when governments will not allow refugees to access national systems and it is considered financially viable. In general, refugees should be provided with a similar level of services to that of the “average” national [2]. In most countries where refugees are located, it is unlikely that the “average” national can afford private health insurance.

#### Microinsurance and community-based health insurance schemes

The terms “microinsurance” and “community-based health insurance” (CBHI) are often used interchangeably; however, microinsurance is a broader concept that includes CBHI schemes. Microinsurance refers to public, private, not-for-profit, or community-based insurance schemes whose services operate at the local level and are specified to the needs of the poor. It targets those who would generally be excluded from mainstream insurance coverage. It protects the vulnerable from risks specific to their situation (e.g., flooding, catastrophic health expenditures) based on the risk likelihood and cost. Individuals pay low premiums to a small pool, and the fund provides limited coverage with a small but still meaningful payout. Microinsurance schemes are often integrated into existing social protection systems [11].

Challenges arise when enrolling the extremely poor who generally cannot pay into the pool, which means that subsidization is required. Microinsurance schemes can also be difficult to sustain, particularly for health, as they require individuals to consistently pay into the pool, which may collapse if too many people withdraw at once.

CBHI is a microinsurance scheme focused on mitigating health risks. It is managed at the community level by a community organization rather than a public, private, or not-for-profit group. The community organization collects premiums and pools funds to protect enrolled community members from risks. Enrollment is voluntary, and usually these schemes emerge when the social protection system or private sector cannot reach affected individuals. CBHI generally has low transaction costs and high trust but, like microinsurance, struggles with maintaining enrollment and creating a large enough pool to adequately cover multiple claims at once [10, 11].

To our knowledge, CBHI has not yet been implemented to scale for refugees in LICs or MICs. We recommend that, together with traditional health insurance for refugees in protracted settings, CBHI for refugees in similar settings should be explored. It can substitute, complement, link with, supplement, or provide an alternative for other refugee health care mechanisms.

#### Combined indexed insurance and catastrophe bonds (Refugee Health Financing Model)

Insurance can operate at several levels in emergency-prone contexts, offering disbursements to states, organizations, communities, or individuals. Regional risk-transfer and insurance mechanisms for natural disasters have existed for more than a decade and are increasingly being explored for other crises, such as pandemics. Mutualizing risk shares costs associated with loss and risk among many parties, so no single party is solely responsible. Governments, businesses, communities, or multi-lateral agencies can pool funds to protect populations against disasters, linking payment to natural disasters and now epidemics [5, 6].

Bonds are a common capital market tool where a creditor loans money to a public, corporate, or other entity, which issues them a bond. The bond lasts until a preset date (maturity date), and once mature, the loaned funds (bond principal) are returned. Interest is usually paid out periodically until maturity. Bonds have either a set or variable interest rate (coupon). Catastrophe bonds are issued by a public entity, insurance company, or other organization to an investor. They have a high coupon rate, usually to reinsure another party. If a catastrophe occurs (currently, most of these bonds are for natural disasters), the investor defers or forfeits payment of the interest and/or principal. Instead the money is used to address the catastrophe. If there is no catastrophe, the bonds typically mature within 3 years, and investors are paid back the principal with interest [5].

Three different examples are presented (Box 2) that could be adapted and explored for different refugee contexts: (1) the African Risk Capacity (ARC) group [12], (2) the Caribbean Catastrophe Risk Insurance Facility (CCRIF) [13], and (3) the Pandemic Emergency Financing Facility (PEF) [14]. Could such a combination of modified risk-transfer instruments be used for refugee emergencies pre-crisis? The answer is unknown.

**Fig. 1:**
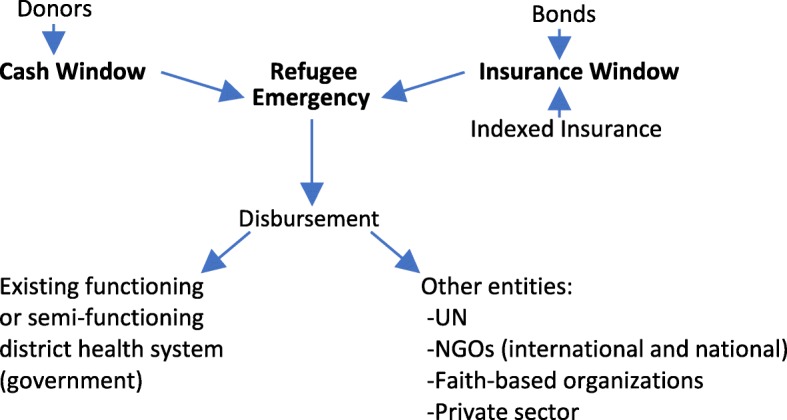
Refugee Health Financing Model (FinRef)

We present here a model modified from the PEF called the Refugee Health Financing Model (FinRef), which requires further exploration by a multi-disciplinary team including experts in finance, insurance, health, development, and humanitarian emergencies (Fig. 1).

The objective of FinRef is to provide funding from diverse sources, using a variety of financing mechanisms, to provide health services to refugees during the acute phase of an emergency using pre-emergency planning. A cash window for immediate funding uses existing pool funding mechanisms that sets aside donations to be immediately available for emergency responses to humanitarian crises. Funding is channeled through UN-managed humanitarian pooled funds, such as the global Central Emergency Response Fund (CERF) and Country-Based Pooled Funds (CBPFs). These pool funding modalities have almost doubled over the past decade and reached $1.2 billion in 2016 [6]. Despite this significant increase, the CERF and CBPFs are limited, available for all sectors, and cannot sufficiently address the health needs of refugees.

An insurance window could be funded by *bonds* financed by the private sector or multi-lateral organizations and have clear parametric indices for payout. For bonds, there are at least two alternatives: (1) Short-term bonds are meant to bridge a gap due to insufficient funds at the beginning of an emergency. Guarantees from multi-lateral agencies or governments to repay the bond at a specified later date could be provided to reduce risk. However, with this mechanism, funds from different and/or more traditional sources would have to be found to eventually pay back the bond holders; and (2) Longer-term bonds with their implicit risk could be issued with no guarantee of repayment of the principal. These bonds would have higher yields than the short-term bonds previously discussed.

The *indexed insurance* window could consist of insurance financed by the private sector, multi-lateral and bi-lateral organizations, and UN agencies with clear parametric indices. For example, the UNHCR expends millions of dollars each year on health services for refugees. Some of these funds could be “set aside” for health insurance pre-emergency for host countries. The parametric indices need to be developed and verified, but some examples could include: (1) the Fragile States Index, which is a critical tool that identifies key factors that push a state towards the brink of failure [15], and (2) a specified number of refugees crossing a border. However, considerable analysis needs to occur to decide which, if any, indicators are measurable and predictable. When possible, funds should go to government-level offices that manage health systems and are responsible for integrating refugees. Existing health systems, whether functional or semi-functional, will likely need increased capacity and support from the UN and NGOs. If national health systems at the district level are not functional or cannot sufficiently address the emergency needs of refugees, then other entities should receive the funds. These entities include the UN, international and national NGOs, faith-based organizations, and in some rare circumstances the private sector (e.g., mostly privatized health systems, such as the one in Lebanon). As with all financial instruments, there are potential benefits as well as risks that must be examined according to context. For FinRef specifically, issues such as market speculation must be considered, particularly in a humanitarian context where refugees are so reliant upon external support. Since the objective is to have refugees integrated into national health systems with funds used to improve the system for both nationals and refugees, incentives and agreements should be put into place with these entities to ensure that once the situation is more stable, refugees and the funding will move from these “parallel” systems to national systems. Doing so will require building capacity of the latter.

### Grants and loans to refugee-hosting governments

The humanitarian-development nexus has been recognized as a major challenge for decades [4]. How can humanitarian and development actors work with governments to implement resilient and integrated programs that will benefit nationals and refugees? In 2016, the World Bank established a $2 billion window under international development assistance (IDA) to support refugee-hosting LICs. Such a new window was justified because LICs rarely, if ever, use their own scarce resources to cover non-nationals. IDA countries that host more than 25,000 refugees or have a population that is more than 0.1% refugees can access these funds. Countries will submit a forced displacement strategy note that explains how these funds will strategically be used to support their citizens and refugees.

In 2016, the World Bank also launched the Global Concessional Financing Facility (GCFF), which provides financial support to MICs addressing humanitarian crises. While relying on grants from donor countries, it leverages its money to yield four times the amount for concessional financing through long-term loans with low interest. The GCFF will expand to a global scale (Box 3) [16]. Further research is needed to show that refugees together with the assistance that comes with them help to improve the host economies [4].

Grants and low-interest, long-term concessional loans to LICs and MICs hosting refugees are a new financial tool to incentivize host governments to develop strategies that take into account the needs of their nationals as well as refugees. In terms of health services for refugees, it is likely that such financing mechanisms will be mostly used in post-emergency protracted settings for refugees in and outside of camps. We would advocate that the objective of such grants and loans should be to integrate health services for refugees into national health systems when they are functioning and to improve semi-functioning national health systems; in both scenarios, these funds should ultimately improve health services for nationals while allowing the refugees to benefit as well.

There are many refugee camps throughout the world that continue to provide parallel health services to refugees. Some are in remote areas, while others are near more populated locales. The UNHCR continues to fund these parallel health services, primarily through international and national NGOs. For the most part, refugees have limited or no livelihoods in these camps, and thus health services remain free of charge. In these protracted refugee camps, mortality rates are generally lower and maternal-child health outcomes are generally better compared with those of the host country nationals [17, 18]. Furthermore, surrounding host nationals often use the health services provided in these camps. We recommend that these parallel health services in protracted refugee camps end, and that the funding for these services be used to improve the national health systems while allowing refugees to access such systems—again, only when such national systems exist or could be improved to be functional. There is the possibility that the quality of services that the refugees receive in these protracted camp settings would be lower in the national systems. However, equity and social cohesion aspects need to be considered, which is why a UNHCR principle is to provide a level of services to refugees that is similar to that received by nationals in that area [2]. There will likely be many challenges in moving from parallel to integrated services in these protracted refugee camps. Beyond political complications, an initial injection of funds may be necessary to allow the transition to succeed.

### Role of remittances

Numerous health access and utilization surveys have documented that refugees pay out-of-pocket expenses for their health care, particularly those refugees outside of camps. A rather extreme example is in Jordan, where the non-Syrian refugees pay expensive non-Jordanian health care rates compared to the Syrian refugees. In 2016, 44% of the interviewed non-Syrian refugee households spent an average of Jordanian dinar (JOD) 116.9 (43%) on health care during the last month of the interview, although their combined monthly income is JOD 273.4 [19]. In many countries, refugees are not officially allowed to work, and thus they earn their money from unofficial work, borrowing, and remittances.

Remittances are an important part of money flow and revenue globally. For example, economic migrants are now sending earnings to families and friends in developing countries at levels above $441 billion, which is three times the volume of official aid flows. Remittances constitute more than 10% of the gross domestic product (GDP) in approximately 25 developing countries, and they increase investments in health, education, and small businesses in various communities [20]. Research on remittances during refugee contexts is scarce, but it is assumed that they have a positive impact on the wellbeing of those receiving them [21, 22]. Remittances may help refugees pay for user fees or medicines, but they should not be relied on as a substitute for health financing. Rather, facilitating remittances can complement these initiatives.

The role of remittances in refugee settings needs to be better understood, and certain actions should be explored to make remittances flow more fluidly and efficiently in such settings. These include exploring methods to reduce or eliminate surcharges specific to refugees, working with remittance agencies to ensure that certain types of refugee identification are accepted, and working with specific countries to develop national policies that facilitate the sending and receiving of remittances.

## Innovative method of purchasing projects and services: pay for performance

Pay for performance (P4P) is an umbrella term for financing initiatives aimed at improving the quality, efficiency, effectiveness, and the overall value of health services. It shifts financial risk from a traditional funder, usually a government, to a new investor who provides upfront capital to scale an evidence-based program to improve outcomes. Targets are set for service providers to achieve. Achieving these targets not only improves service delivery but should also reduce the costs, and the savings generated are then used by the local government or donor to pay back investors over time. In theory, repayment only occurs if the program is successful, so investors assume the risk. Outcomes are measured according to pre-defined metrics and are verified by an independent agency. The P4P contracts have financing agreements that provide upfront capital to support service delivery throughout the project period. Depending upon who provides the revenue, this could provide much needed funding from non-traditional donors, particularly the private sector. P4P requires time in order to undertake in-depth assessments requiring significant data, set up the financial arrangements, and negotiate among the various partners [23–25].

P4P requires a great deal of preparation, specific data, and measurement of impact indicators that are rarely available at the beginning and early stages of an emergency. Some reviews of P4P have not found significant improvements in health outcomes, and more research is needed on the exact mechanisms through which incentives and ancillary components operate [25, 26]. Furthermore, during the acute phase of an emergency, one generally addresses the health system in a comprehensive manner, which makes it difficult for P4P to be applied. Consequently, we recommend trialing P4P in refugee settings for specific interventions that are relatively easy to measure and where evidence already exists of their efficacy and effectiveness. These include increasing vaccination coverage (measured as fewer measles or cholera outbreaks), improving birth outcomes (measured as deliveries with a skilled birth attendant), and reducing deaths due to a malaria (measured as spraying, bed nets, rapid diagnostic tests, following treatment protocols, etc.). These specific interventions all are possible to implement and measure in protracted refugee settings, particularly in refugee camps. Measurements of numerators and denominators are more easily obtained in camps then in out-of-camp settings, and partners are often international or national NGOs with clear roles and responsibilities. Refugees have fewer choices regarding services in camps than out of camp. Therefore, P4P has an important, but relatively limited role in the delivery of specific health interventions in protracted refugee settings, particularly camps.

At a time when funding is insufficient and being reduced for protracted and forgotten refugee settings, P4P would allow for private sector funds to possibly finance some of these types of interventions. Furthermore, the effectiveness of the service providers to deliver these interventions would increase while the costs may decrease. Therefore, P4P is appropriate in protracted refugee settings, particularly camps, when addressing specific health interventions, but not for broad health systems issues. It is a risk-transfer modality that is dependent upon planning.

## Conclusion

The number and protracted settings of refugees and consequently their health needs are increasing, and there is insufficient funding from HICs to address them. By learning from innovative health funding from other situations and adapting them to refugee-specific contexts, existing funding can be used more effectively, additional funding from non-traditional sources such as the private sector and donors who typically provide to development scenarios can increase, and such funding could benefit national health systems as well as refugees.

Donors, investors, hosting governments, UN agencies, and NGOs will need to be prepared to trial different funding schemes according to different refugee contexts, as many unanswered questions need to be explored. This article provides numerous possibilities of novel and innovative health funding mechanisms for refugees. We hope that it will serve as the basis for numerous and diverse organizations and investors to explore these opportunities.

## Box 1: Refugee health insurance in the Islamic Republic of Iran

The Islamic Republic of Iran and the UNHCR launched the health insurance scheme (HISE) for Afghan refugees in 2011 through a semi-private insurance company, as the government did not allow refugees access to the national system at that time. HISE was made available to registered refugees on an individual and voluntary basis with the overall goal of improving equity and financial access to in-patient services, with a special focus on vulnerable populations. The launching of the HISE also aimed at generating additional opportunities for further improvement of refugees’ access to health care and creating a positive impact on their health status. Through minimizing the financial burden of vulnerable refugees, the HISE also aimed at indirectly generating positive impacts on the prevention of gender-based violence, school drop-outs, and other issues. The scheme provided complementary health insurance coverage to 331,003 Afghan refugees, including 214,652 vulnerable persons and 116,351 non-vulnerable refugees. Registered refugees in Iran have the possibility to obtain work permits and thus livelihoods. This allowed some of them to pay for their premiums and co-payments themselves. For those who could not pay and met the vulnerability criteria, the UNHCR covered their costs. In 2015, negotiations were concluded with the government to allow refugees access to the national HISE.

## Box 2: Examples of indexed insurance and catastrophe bonds

*The African Risk Capacity (ARC) group*. The ARC group holds governments accountable for mitigating natural disasters and guaranteeing quick service delivery. ARC is Africa’s first sovereign catastrophe insurance pool. It uses data that combines weather and crop data with information on vulnerable populations and past analysis of the costs of response. Disbursements to ARC policy-holding governments are triggered when the estimated cost of responding crosses an agreed-upon pre-defined threshold. Since its launch in May 2014, nine countries have joined the ARC, and three participating countries (Mauritania, Niger, and Senegal) have received their first payouts, totaling a combined US$26 million.

*The Caribbean Catastrophe Risk Insurance Facility (CCRIF)*. The CCRIF offers insurance coverage to Caribbean governments for natural disasters, combining it with capital market instruments and a parametric index. It covers 17 countries for earthquakes, tropical cyclones, and excessive rainfall. Countries purchase insurance through an annual premium, and are insured for up to $100 million. If an event occurs, disbursements occur within 2 weeks. The CCRIF uses segregated portfolios to manage risk while maintaining a single operational structure. In addition to offering insurance, the CCRIF finances itself through the reinsurance market, catastrophe bonds, and catastrophe swaps. To reinsure the CCRIF, the World Bank issued $30 million in catastrophe bonds. The bonds rely on a parametric index that can be triggered annually, and they cover some of the risk from storm surges, wind from tropical cyclones, and earthquakes. If the trigger occurs, then the principal is reduced (by preset terms) and paid to CCRIF. The investors are private funders and companies that can trade these bonds on secondary markets.

*Pandemic Emergency Financing Facility (PEF)*. The PEF is an innovative insurance-based mechanism created by the World Bank and its partners that will provide surge funding in the form of grants to LICs to respond to rare, high-severity disease outbreaks on the regional level to attempt to prevent them from becoming pandemics. The PEF is needed because there is no fast-disbursing financing mechanism to provide significant funds to resource-constrained countries early enough to support them to combat an escalating epidemic. The PEF includes insurance, bond, and cash windows. The insurance window covers a maximum amount of US$500 million over 3 years through catastrophic (pandemic) bonds and pandemic insurance. Payment is trigged by an outbreak of specific diseases or disease families with pandemic potential. Each disease has a maximum insurance coverage per event. To provide coverage, both premiums and bond coupons are paid by development partners. If there is a catastrophe, then funding is released according to parametric indices that are based on epidemic size, severity, and spread, and have verified action criteria. The cash window covers a maximum amount of US$100 million that is replenished annually through donors. It complements the insurance window by (1) providing supplemental financing for addressing pathogens covered by insurance, (2) covering severe outbreaks not included in the insurance scheme, and (3) acting as a conduit for efficient and effective surge financing during the epidemic for development partners. The PEF funds are provided to two types of responders: (1) national entities (e.g., Ministries of Health) and (2) accredited international organizations and NGOs.

## Box 3: The Global Concessional Financing Facility

The Global Concessional Financing Facility (GCFF) provides financial support to MICs impacted by refugee crises across the globe. It bridges the gap between humanitarian and development assistance and strengthens the resilience of countries impacted by refugee crises by assisting both host communities and refugees. It supports policy reforms and programs in areas such as education, health, and job creation to create sustainable development outcomes.

The GCFF builds upon the Concessional Financing Facility for the Middle East and North Africa, expanding it globally to MICs. Both are part of the World Bank’s Global Crisis Response Platform, which responds to crises by combining knowledge, resources, and financial tools in a manner that emphasizes systematic, scaled-up support.

The GCFF relies on grants from donor countries, but it leverages every dollar to yield four times the amount for concessional financing (long-term loans with low interest). The project facilitates the coordination among humanitarian agencies and development banks, so that they respond to refugee emergencies together. Its current goal is to raise US$1 billion in grants for Jordan and Lebanon, as well as US$500 million in grants for other MICs, during the next 5 years. In doing so, the GCFF would generate US$6 billion as concessional financing.

## Abbreviations

ARC: African Risk Capacity (group)
CBHI: Community-based health insurance
CCRIF: Caribbean Catastrophe Risk Insurance Facility
FinRef: Refugee Health Financing Model
GCFF: Global Concessional Financing Facility
GDP: Gross domestic product
HIC: High-income country
HISE: Health insurance scheme
IDA: International development assistance
JOD: Jordanian dinar
LIC: Low-income country
MIC: Middle-income country
NGO: Non-governmental organization
P4P: Pay for performance
PEF: Pandemic Emergency Financing Facility
UN: United Nations
UNHCR: United Nations High Commissioner for Refugees
